# Effect of Weight Loss, Exercise, or Both on Undercarboxylated Osteocalcin and Insulin Secretion in Frail, Obese Older Adults

**DOI:** 10.1155/2017/4807046

**Published:** 2017-08-23

**Authors:** Georgia Colleluori, Nicola Napoli, Uma Phadnis, Reina Armamento-Villareal, Dennis T. Villareal

**Affiliations:** ^1^Division of Endocrinology, Diabetes, and Metabolism, Baylor College of Medicine, Houston, TX 77030, USA; ^2^Center for Translational Research on Inflammatory Diseases (CTRID), Michael E. DeBakey VA Medical Center, Houston, TX 77030, USA; ^3^University Campus-Biomedico, 00128, Rome, Italy

## Abstract

**Background:**

Obesity exacerbates age-related decline in glucometabolic control. Undercarboxylated osteocalcin (UcOC) regulates pancreatic insulin secretion. The long-term effect of lifestyle interventions on UcOC and insulin secretion has not been investigated.

**Methods:**

One hundred seven frail, obese older adults were randomized into the control (*N* = 27), diet (*N* = 26), exercise (*N* = 26), and diet-exercise (*N* = 28) groups for 1 year. Main outcomes included changes in UcOC and disposition index (DI).

**Results:**

UcOC increased in the diet group (36 ± 11.6%) but not in the other groups (*P* < 0.05 between groups). Although similar increases in DI occurred in the diet-exercise and diet groups at 6 months, DI increased more in the diet-exercise group (92.4 ± 11.4%) than in the diet group (61.9 ± 15.3%) at 12 months (*P* < 0.05). UcOC and body composition changes predicted DI variation in the diet group only (*R*^2^ = 0.712), while adipocytokines and physical function changes contributed to DI variation in both the diet (∆*R*^2^ = 0.140 and 0.107) and diet-exercise (∆*R*^2^ = 0.427 and 0.243) groups (*P* < 0.05 for all).

**Conclusions:**

Diet, but not exercise or both, increases UcOC, whereas both diet and diet-exercise increase DI. UcOC accounts for DI variation only during active weight loss, while adipocytokines and physical function contribute to diet-exercise-induced DI variation, highlighting different mechanisms for lifestyle-induced improvements in insulin secretion. This trial was registered with ClinicalTrials.gov number NCT00146107.

## 1. Introduction

The role of bone as an endocrine organ able to regulate energy metabolism has emerged during the past years [1, 2]. The crosstalk between bone, pancreas, and adipose tissue is mediated not only by insulin and adipocytokines but also by osteocalcin (OC), an osteoblast-secreted protein able to act as an endocrine mediator in its undercarboxylated form (UcOC). In conditions of bone resorption, the acidic environment favors OC undecarboxylation and UcOC release into the circulation where it exerts its hormonal effect enhancing insulin secretion, sensitivity, *β*-cell proliferation [1–3], and muscle's response to acute exercise [4]. The observation of UcOC's effects on energy metabolism and glucometabolic control in animal models has raised the interest towards its potential in humans. Lower OC and UcOC levels were related to higher fasting glucose and fat mass in different populations [5, 6], while higher serum UcOC levels were associated with lower risk of developing type 2 diabetes (T2D) [7].

Frailty is an age-related condition characterized by increased inflammation and oxidative stress, which is aggravated by obesity [8–10]. Furthermore, the age-related decline in metabolic control is exacerbated by obesity which has developed into an epidemic in the Western world. By 2030, 20% of the population will be represented by older adults (defined as age ≥ 65 years) of whom half will be obese [11]. Lifestyle modifications consisting of diet and regular exercise are the cornerstone of treatment for obesity and T2D. Even if the adoption of a lifestyle strategy in frail older adults is still under debate [12], its ability to improve insulin sensitivity is well documented [9, 13] whereas its capacity to improve insulin secretion is still unexplored. Exercise and diet are known to induce changes in body composition, adipocytokines, and bone mineral density (BMD), all of which have been shown to correlate with UcOC [1, 2, 6]. For example, osteocalcin was inversely related to fat mass and leptin in both mice and humans [1, 5, 6, 14], a correlation independent of other markers of bone formation [5, 6]. In addition, weight loss and associated changes in body composition are known to increase bone turnover [15, 16], which is the main trigger of UcOC release. Furthermore, a direct impact of acute exercise on circulating UcOC has been proposed [14, 17–19], and some authors hypothesized that improvement in metabolic parameters due to weight loss or exercise-induced weight loss could be mediated by UcOC [1, 14]. To our knowledge, the long-term effect of different lifestyle interventions on circulating UcOC and insulin secretion has not been investigated.

The objective of this study was to compare the long-term effects of different lifestyle interventions (diet, exercise, and the combination of both) on circulating UcOC and DI (disposition index: an index of insulin secretion after correction for insulin resistance) [20] and their correlations in a population of frail, obese older adults. We hypothesized that diet-induced weight loss would increase UcOC through an increase in bone resorption and that this effect would contribute to glucometabolic control through an increase in insulin secretion. On the other hand, we hypothesized that chronic exercise, which reduces bone turnover, would not affect UcOC.

## 2. Materials and Methods

This is a secondary analysis of a randomized control trial (RCT) conducted on frail, obese older adults, investigating the independent and combined effect of diet and exercise on physical function. The primary results showed that diet and exercise can independently improve physical function and ameliorate frailty; however, the combination of both interventions provides greater improvement in physical function and frailty than either of them alone [9]. The current study reports a secondary analysis of the RCT examining changes in UcOC and DI.

### 2.1. Study Population

This RCT was approved by the institutional review board and monitored by an independent data and safety monitoring board. The study population, described elsewhere [9], was recruited from the community through advertisements. All subjects provided a written informed consent for participation; inclusion criteria were the following: aged ≥ 65 years; body mass index ≥ 30 kg/m^2^; sedentary lifestyle (regular exercise of <1 h per week) during the previous 6 months; stable body weight (within 2 kg) over the preceding year; stable medications for at least 6 months before enrollment; mild-to-moderate frailty determined by meeting at least two out of three operational criteria: modified physical function test score of 18–32, peak oxygen uptake (VO_2peak_) of 11–18 ml·kg^−1^ per minute, or difficulty in performing two instrumental activities of daily living (ADL) or one basic ADL [9, 10, 21]. Exclusion criteria were the following: musculoskeletal/neuromuscular impairments that precluded exercise training; severe cardiopulmonary disease; significant cognitive impairment; history of malignancy; history of diabetes or fasting glucose of ≥126 mg·dl^−1^ [22]; and current smoking.

### 2.2. Study Design

Participants (*n* = 107) were randomly assigned to one of four groups stratified for sex: (1) control group, (2) 10% diet-induced weight-loss group (diet group), (3) exercise training without weight loss (exercise group), and (4) 10% diet-induced weight loss and exercise training (diet–exercise group) and followed for 1 year. As described previously [9], participants in the control group met monthly with the staff to receive general information regarding a healthy diet and were asked not to participate in weight loss or exercise program. Participants in the diet group met weekly with an experienced dietitian and were prescribed a balanced diet that provided a 500–750 kcal/day deficit and contained 1 g/kg/body weight of high-quality protein [12]. The goal of the diet program was a weight loss of ~10% from baseline to 6 months, followed by maintenance of the achieved weight for the remaining 6 months of the study. Standard behavioral strategies to modify eating habits were adopted at the weekly visit, during which the dietitian reinforced dietary compliance. Participants in the exercise group were counseled on maintaining a weight-stable diet. They participated in ~90 min thrice-weekly multicomponent exercise sessions comprised of 15 min flexibility exercise, 30 min aerobic exercise, 30 min resistance exercise, and 15 min balance, which were supervised by a physical therapist at our exercise center. Aerobic exercises consisted of treadmill, stair climbing, and stationary cycling. Participants exercised at 65% of peak heart rate which was gradually increased to 70–85% of peak heart rate. Resistance exercises consisted of nine upper and lower extremity exercises using weight-lifting machines. The flexibility exercises included lateral trunk and flexion exercises while the balance exercise included trunk rotation exercises performed in increasing difficult stances [9]. The initial sessions were 1-2 sets of 8–12 repetitions at 65% of the one-repetition maximum which was gradually progressed to 2-3 sets at ~85% of the one-repetition maximum [9]. Participants in the diet-exercise group participated in both the weight loss and exercise programs described above. All subjects were provided supplements to ensure an intake of 1500 mg/d of calcium and 1000 IU/d of vitamin D [9]. Further details of the interventions including compliance data and exercise adaptations have been reported previously [9].

### 2.3. Outcome Measures

The outcomes in this secondary analyses were changes in UcOC and DI at 12 months. Other outcomes included oral glucose tolerance test (OGTT) variables, adipocytokines, bone turnover markers, body composition, and bone mineral density (BMD), muscle strength, and peak oxygen consumption (VO_2peak_). Assessors were blinded to group assignments.

### 2.4. Oral Glucose Tolerance Test

A standard 75 g OGTT was performed after an overnight fast. Glucose and insulin were measured from venous blood samples obtained in fasted state, 30, 60, 90, and 120 min after glucose ingestion using glucose oxidase method (YSI Inc., Yellow Springs, OH, USA) and radioimmunoassay. Insulin sensitivity index (ISI) was calculated using the following formula: 10000/square root of [(fasting glucose × fasting insulin) × (mean glucose × mean insulin during OGTT)] [23]. The ISI is correlated (*r* = 0.73) with the whole body glucose disposal rate during a euglycemic insulin clamp study [23]. To minimize the acute effects on glucoregulation, the OGTT was performed ~72 h after the last exercise. Insulinogenic index (IGI) was calculated using the following formula: (insulin at 30 minutes − fasting insulin)/(glucose at 30 minutes − fasting glucose) [24]. DI was calculated by multiplying the IGI by the ISI to determine whether insulin secretion was appropriate for the degree of insulin resistance in accordance with Bergman et al. [20]. By correcting for the hyperbolic relationship between insulin secretion and insulin sensitivity, the DI is an accurate measurement of *β*-cell function [20]. A low DI is an early marker of inadequate *β*-cell compensation which predicts future diabetes [25].

### 2.5. Fasting Blood Analyses

Serum samples were collected after at least 40 h (up to 72 h) from the last bout of exercise in order to exclude its acute effect on circulating markers [26]. UcOC was directly measured using an enzymatic immunoassay that uses two monoclonal antibodies that are highly specific for UcOC (Takara Bio USA Inc., Mountain View, CA). High-sensitive C-reactive protein (hs-CRP) was measured using immunoturbidimetric assay (Hitachi 917, Indianapolis, IN, USA). Total osteocalcin (Metra OC; Quidel, San Diego, CA), C-terminal telopeptide of type 1 collagen (CTX) (Crosslaps; Nordic Bioscience Diagnostics, Herlev, Denmark), soluble tumor necrosis factor receptor 1 (sTNF R1) (R&D, Minneapolis, MN, USA), adiponectin (Linco, St. Louis, MO, USA), and interleukin 6 (HS600B; R&D Systems, Minneapolis, Minnesota, USA) were also measured using enzymatic immunoassays. Leptin (Leptin HL 81K; Linco Research Inc.), intact N-terminal propeptide of type 1 procollagen (P1NP) (Orion Diagnostica, Espoo, Finland), and insulin (Linco Research, St. Louis, MO) were measured using radioimmunoassays. The coefficient of variation for these measurements was less than 10%.

### 2.6. Body Composition and BMD

Fat mass (FM), fat-free mass (FFM), trunk fat mass, and whole body BMD (WB BMD) were measured with the use of the dual-energy X-ray absorptiometry (Delphi 4500/w, Hologic) as described previously [27].

### 2.7. Physical Function

VO_2peak_ was assessed during graded treadmill walking by indirect calorimetry (True Max 2400; ParvoMedics), as described previously [10]. Briefly, the speed was adjusted to identify the fastest comfortable walking speed for the subject during the 3–5 minutes of warm up on the treadmill at 0% grade. While the speed was held constant during the test, the elevation was progressively increased by 2 to 3% every 2 minutes. Patients were allowed to lightly hold on to a handrail to maintain their balance during the test. Cardiorespiratory data were collected using a computerized system every 30 seconds. The test was terminated when the participant became too fatigued to continue. Isokinetic knee extensor (KE) and knee flexor (KF) strength were evaluated using a dynamometer (Biodex System 3 dynamometer Shirley, NY) as described previously [28]. Subjects were seated with their backs supported and hips placed at 120° of flexion. Tests were performed at 60° per second, while for the isometric test, the arm of the dynamometer was fixed at 45° of flexion.

### 2.8. Statistical Analysis

The sample size calculated for the main outcome of this study was sufficient to detect a difference of 1.4 ± 2.6 (SD) in the change in UcOC among the groups, at an alpha level of 5%. Intention-to-treat analyses were performed using SAS version 9.3 (SAS, Cary, NC, USA). Analyses of variance (ANOVA) or chi-square tests were used to compare baseline characteristics of the population. Mixed model repeated measures ANOVA was used to test longitudinal changes between groups, adjusting for baseline values and sex. Within the framework of the mixed model, when the *P* value for an interaction was significant, prespecified contrast statements were used to test three hypotheses: changes in the diet group were different from those in the control group; changes in the exercise group were different from those in the control group; and changes in the diet-exercise group were different from those in the diet group and exercise group. Analyses for within-group changes were performed using mixed model repeated measures ANOVA.

Hierarchical multiple regression was used to test the effect of bone turnover on UcOC variation in the diet and diet-exercise groups, adjusting for other variables affecting UcOC including body composition and circulating adipocytokines. The following models were used: model 1: bone profile changes (P1NP, CTX, and WB BMD); model 2: model 1 plus body composition changes (FFM, FM); and model 3: model 2 plus adipocytokines changes (IL6, CRP, sTNF R1, adiponectin, and leptin). Hierarchical multiple regression was also used to test the effect of UcOC on DI variation in the diet and diet-exercise groups, adjusting for other variables affecting DI including body composition, adipocytokines, and physical function. The following models were used: model 1: UcOC changes; model 2: model 1 plus body composition changes; model 3: model 2 plus adipocytokines changes; and model 4: model 3 plus physical function changes (VO_2peak_, KE strength, and KF strength).

Sensitivity analyses that validated the statistical results obtained included multiple imputation for missing fitness data (which confirmed a similar pattern of results). Data are presented as least-square adjusted means (SE) from the repeated measures analyses, unless otherwise specified. Statistical tests were two tailed, and *P* < 0.05 was considered significant.

## 3. Results

### 3.1. Study Population

The results of randomization, follow-up, and compliance have been reported [9]. Briefly, of the 107 participants randomized, 93 (87%) completed the intervention. Fourteen participants (4 in the control group, 3 in the diet group, 4 in the exercise group, and 3 in the diet-exercise group) did not complete the intervention for medical or personal reasons but were included in the intention-to-treat analyses. Baseline characteristics including body composition, bone turnover and BMD, physical function, and adipocytokines did not significantly differ among the groups (Table 1).

As reported, body weight decreased similarly in the diet group (−9.6 ± 1.2%) and diet-exercise group (−9.4 ± 0.8%), while weight was stable in the exercise group (−0.6% ± 0.7%) and control group (−0.2% ± 0.7%) [9].

### 3.2. Undercarboxylated Osteocalcin

**Figure 1:**
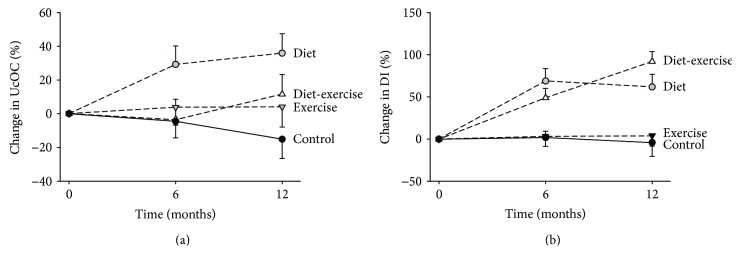
Mean percent changes in undercarboxylated osteocalcin (UcOC) (a) and disposition index (DI) (b) during the 1-year interventions. In (a), the changes in UcOC in the diet group differed significantly from the changes in UcOC in the diet-exercise, exercise, and control groups. In (b), the changes in DI in the diet-exercise group differed significantly from the changes in DI in the diet, exercise, and control groups. I bars indicate standard errors.

Serum UcOC increased in the diet group at 6 months (29.2 ± 11.0%) and remained elevated at 12 months (36.0 ± 11.6%); in contrast, serum UcOC did not change in the control group, exercise group, and diet-exercise group (*P* = 0.04 for the between-group differences) (Table 2, Figure 1(a)). Total serum OC increased in the diet group (21.8 ± 7.7%) whereas it decreased in the exercise group (−14.5 ± 6.3%); in contrast, total osteocalcin did not change in the diet-exercise and control groups as previously reported (*P* < .001 for the between-group differences) (Table 2) [15]. UcOC/total OC ratios did not change in any of the intervention groups (Table 2).

### 3.3. Insulin Secretion

The IGI did not change in any of the intervention groups (Table 2). On the other hand, although the ISI increased similarly in the diet-exercise and diet groups at 6 months, the ISI increased more in the diet-exercise group than in the diet group at 12 months (87.6 ± 19.2% versus 70.0 ± 18.5%; *P* = 0.02) (Table 2). No changes in ISI occurred in both the exercise and control groups, as previously reported [13]. Accordingly, the DI, which adjusts insulin secretion to changes in insulin sensitivity, increased similarly in the diet-exercise and diet groups at 6 months while the DI increased more in the diet-exercise group than in the diet group at 12 months (92.4 ± 11.4% versus 61.9 ± 15.3%; *P* = 0.04) (Table 2, Figure 1(b)).

### 3.4. Body Composition, Bone Turnover and BMD, Physical Function, and Adipocytokines

The effects of the intervention on the following outcomes (except for leptin) were presented in earlier papers and are provided again to assist with the interpretation of the present report. FFM decreased less in the diet-exercise group (−3.2 ± 0.6%) than in the diet group (−5.3 ± 0.7%), while it increased in the exercise group (2.4 ± 0.5%) [9]. FM and trunk fat decreased similarly in the diet-exercise group (−16.3 ± 1.8% and −16.7 ± 1.9%, resp.) and diet group (−17.4 ± 2.2% and −16.7 ± 2.4%, resp.) and did not change in the exercise group [9]. Serum PINP and CTX increased in the diet group (8.5 ± 6.2% and 35.8 ± 11.4%, resp.), decreased in the exercise group (−14.6 ± 5.5% and −13.4 ± 7.2%, resp.), and did not change in the diet-exercise group [15]. Hip BMD decreased less in the diet-exercise group (−1.1 ± 0.5%) than in the diet group (−2.6 ± 0.4%), whereas it significantly increased in the exercise group (1.5 ± 1.6%); spine and WB BMD did not significantly change [15]. VO_2peak_, KE strength, and KF strength improved similarly in the diet-exercise group (9.1 ± 1.5%, 20.3 ± 4.3%, and 20.6 ± 6.2%, resp.) and exercise group (8.3 ± 1.8%, 23.4 ± 4.5%, and 25.2 ± 4.3%, resp.) and did not change in the diet group [9, 29]. Serum hs-CRP, sTNF R1, and leptin decreased similarly in the diet-exercise group (−27.3 ± 6.6%, −8.7 ± 2.6%, and −38.4 ± 5.3%, resp.) and diet group (−27.0 ± 8.2%, −6.9 ± 2.3%, and −26.2 ± 6.6%, resp.) [13]. Serum adiponectin increased similarly in the diet-exercise group (33.9 ± 12.8%) and diet group (20.6 ± 5.7%) [13].

### 3.5. Hierarchical Models of Variables Predicting UcOC Changes

#### 3.5.1. Diet Group

Model 1 was significant and showed that changes in the bone profile accounted for 27.6% of the variance in UcOC (*R*^2^ = 0.276, *P* = 0.007) (Table 3). Model 2 accounted for 43.4% of the variance in UcOC (*R*^2^ = 0.434, *P* = 0.001) after controlling for changes in body composition; changes between model 1 and model 2 were significant (∆*R*^2^ = 0.158, *P* = 0.013). Model 3 accounted for 71.9% of UcOC variation (*R*^2^ = 0.719, *P* < 0.001) after controlling for body composition and cytokines; changes between model 2 and model 3 were significant (∆*R*^2^ = 0.284, *P* = 0.001). These data suggested that changes in the bone profile are independent predictors of UcOC variation in the diet group but that also changes in body composition and adipocytokines play a significant role.

#### 3.5.2. Diet-Exercise Group

Model 1 was significant and showed that changes in the bone profile accounted for 49.8% of the variance in UcOC (*R*^2^ = 0.498, *P* < 0.001) (Table 3). Model 2 accounted for 50.1% of the variance in UcOC (*R*^2^ = 0.501, *P* < 0.001) after controlling for changes in body composition; changes between model 1 and model 2 were not significant (∆*R*^2^ = 0.003, *P* = 0.867). Model 3 accounted for 61.3% of UcOC variation (*R*^2^ = 0.613, *P* < 0.001) after controlling for body composition and adipocytokines; changes between model 2 and model 3 were not significant. These data suggested that changes in the bone profile are independent predictors of UcOC variation in the diet-exercise group and that changes in body composition and adipocytokines play only a minor role.

### 3.6. Hierarchical Models of Variables Predicting DI Changes

#### 3.6.1. Diet Group

Model 1 was significant and showed that UcOC accounted for 20.2% of the variance in DI, (*R*^2^ = 0.202, *P* = 0.005) (Table 4). Model 2 accounted for 71.2% of the variance in DI (*R*^2^ = 0.712, *P* < 0.001) after controlling for changes in body composition; changes between model 1 and model 2 were significant (∆*R*^2^ = 0.511, *P* < 0.001). Model 3 accounted for 81.0% of UcOC variation (*R*^2^ = 0.810, *P* = <0.001) after controlling for body composition and adipocytokines changes; changes between model 2 and model 3 were significant (∆*R*^2^ = 0.140, *P* = 0.001). Model 4 accounted for 93.5% of DI variation (*R*^2^ = 0.935, *P* < 0.001) after controlling for body composition, adipocytokines, and physical function changes; changes between model 3 and model 4 were significant (∆*R*^2^ = 0.083, *P* < 0.001). These data suggested that UcOC is an independent predictor of DI but that body composition, adipocytokines, and physical function changes also contribute to DI variation in the diet group.

#### 3.6.2. Diet-Exercise Group

Model 1 was not significant and showed that UcOC accounted for only 7.8% of the variance in DI (*R*^2^ = 0.078, *P* = 0.101 according to Table 4). Model 2 was statistically significant and accounted for 27.6% of the variance in UcOC (*R*^2^ = 0.276, *P* = 0.013) after controlling for changes in body composition; changes between model 1 and model 2 were significant (∆*R*^2^ = 0.198, *P* = 0.018). Model 3 accounted for 70.2% of UcOC variation (*R*^2^ = 0.702, *P* < 0.001) after controlling for body composition and adipocytokines; changes between model 2 and model 3 were significant (∆*R*^2^ = 0.427, *P* < 0.001). Model 4 accounted for 94.6% of DI variation (*R*^2^ = 0.946, *P* < 0.001) after controlling for body composition, adipocytokines, and physical function; changes between model 3 and model 4 were significant (∆*R*^2^ = 0.243, *P* < 0.001). These data suggested that UcOC and body composition changes are not predictors of DI variation, whereas adipocytokines and physical function changes contribute to DI variation in the diet-exercise group.

## 4. Discussion

Our RCT of lifestyle interventions in obese older adults showed that UcOC increased after 6 months of diet and remained elevated thereafter but not after 6 or 12 months of exercise or diet-exercise. On the other hand, insulin secretion as assessed by the DI increased after 6 months of either diet or diet-exercise, but DI continuously increased after 12 months only after diet-exercise. In our study, UcOC and body composition were independent predictors of DI in the diet group only, while adipocytokines and physical function contributed to DI prediction in both the diet and diet-exercise groups.

During the past decade, the view of the skeleton as a metabolically inactive tissue, whose main roles are protection of internal organs, support for locomotion, and host of hematopoiesis, has completely changed. The studies of Lee et al. shed light on bone's novel function as a metabolic regulator, in which osteocalcin's action seems pivotal [1]. Osteocalcin, an osteoblast-secreted protein involved in bone remodeling, is characterized as having a high affinity for hydroxyapatite in its carboxylated form, but not when undercarboxylated [1–3]. In the acidic environment typical of bone resorption, OC is converted into UcOC and released in the circulation where it may exert its hormonal action [1–3]. Among UcOC targets are the pancreas and adipose tissue, where its ability to promote insulin and adiponectin secretion, as well as pancreatic *β*-cell proliferation, has been documented in animal models and humans [5, 6].

The exponential growth of the obese older population is considered a public health burden especially in light of the associated metabolic consequences [11]. The age-related decline in the pancreatic endocrine function causes the progressive loss of an adequate *β*-cell response to insulin resistance which contributes to glucose homeostasis impairment in obese older adults [30]. Although still questioned by geriatricians because of the weight loss-induced bone and muscle loss, lifestyle intervention is an effective strategy to reduce cardiometabolic risks in obese older adults [13]. However, even if improvements in insulin sensitivity are well documented, the effect of different lifestyle interventions on insulin secretion has not been thoroughly investigated. In fact, even though insulin resistance is commonly associated with obesity and aging, the ability of the pancreas to compensate by increasing insulin secretion determines whether diabetes occurs [31].

Data on the effect of diet and exercise on UcOC are conflicting and no studies have compared the long-term effect of lifestyle interventions in obese older adults. Exercise increased circulating UcOC acutely in middle-aged and younger obese men [14, 17, 19], while 20 weeks of diet or diet plus either vigorous or moderate exercise did not promote any significant change in UcOC in women between 50 to 70 years old [32]. In our group of obese older adults, diet, but not exercise or the combination of both, promoted a significant increase in circulating UcOC at 6 months which remained elevated at 12 months (Figure 1()(a)), leading us to speculate that the increase in the osteoblast-secreted protein occurs only during active weight loss and that it is prevented by the addition of exercise. Considering bone resorption as the main trigger of UcOC release [1, 3, 4], an evidence supported by studies conducted on individuals treated with antiresorptive drugs [33, 34], our results are consistent with our previous findings showing that weight loss-induced increase in bone resorption (and bone loss) is prevented by exercise [15]. In fact, our study showed that bone profile changes accounted for 27.6% of UcOC variance and the inclusion of body composition and adipocytokine changes raised the model predictability up to 71.9%. On the other hand, the addition of body composition and adipocytokine changes did not increase UcOC predictability in the diet-exercise group. It is thus possible that exercise increases UcOC acutely [14, 17, 19], as a consequence of the increase in bone resorption [3, 4] but not chronically as our current data suggest, when bone turnover decreases [15]. As indicated by our data, exercise prevents bone loss and promotes an increase in BMD by inhibiting bone resorption and, consequently, bone turnover, whereas diet has exactly the opposite effect: it increases bone resorption and turnover, ultimately causing bone loss. This finding emphasized the differences between acute and long-term effects of exercise on bone markers [15].

Our exercise group experienced an increase in hip BMD, and the reduction in OC did not correspond to a reduction in UcOC, which did not change. This finding suggests that increased bone resorption promotes the increase in circulating UcOC (as occurred in the diet group) but that the reverse may not be true (i.e., decreased bone resorption may not always decrease UcOC). It is possible that the reduction in UcOC that follows the use of antiresorptive agents [33, 34] represents an extreme artificial scenario, which does not occur with lifestyle modification. The lack of significant changes in UcOc/total OC indicated that the absolute amount, and not the proportion of UcOC relative to total OC, changed with the intervention.

In light of UcOC's ability to improve insulin secretion by *β*-cells [35] and the age-related decline in pancreatic function, we investigated the effect of our interventions on the DI, a measurement of insulin secretion that expresses the ability of the *β*-cells to adequately respond to insulin resistance [20]. Because of the hyperbolic relationship between insulin secretion and sensitivity on the OGTT [20], adjustment of insulin secretion for sensitivity may provide an accurate measurement of *β*-cell function, the bases for which some investigators consider DI a better predictor of T2D compared to insulin sensitivity [36–38]. We previously demonstrated that diet plus exercise improves pancreatic function in obese older adults, increasing DI and insulin clearance, but not affecting the absolute insulin secretion rate, leading to an overall reduction in insulin levels [30]. Accordingly, DI improvements were reported to be proportional to the exercise intensity in a study conducted on prediabetic obese older adults [36]. Nevertheless, the independent and combined effects of diet and exercise on DI in older obese individuals remain unexplored. In the present study, DI improved in the diet group following the same trend of UcOC, whereas the diet-exercise group experienced continuous improvements at the 6-month and 12-month time points (Figure 1()(b)). We hypothesized that DI (and other metabolic improvements as reported [13]) experienced by the diet group might be partially due to the changes in UcOC. Accordingly, UcOC was an independent predictor of DI changes, accounting for 20% of its variation in the diet group but not in the diet-exercise group. Other variables significantly increasing the ability to predict DI in the diet group were the following: body composition, adipocytokines, and physical function, data that suggested that UcOC can mediate DI increase not only through its action on *β*-cells but also indirectly by improving body composition and the inflammatory profile. On the other hand, in the context of the combination of diet and exercise, UcOC and body composition were not significant predictors of DI, while adipocytokines and physical function provided a significant contribution to the model.

Insulin secretion by pancreatic *β*-cells is tightly regulated by several factors including central signals, circulating glucose, insulin, incretins, free fatty acids, and adipocytokines [39]. As the largest insulin-sensitive tissue in nonobese subjects, the skeletal muscle may send signals to the pancreas, which are able to regulate *β*-cell function in an insulin sensitivity-dependent manner [39]. Condition media from insulin sensitivity versus insulin resistant human myotubes (treated with tumor necrosis factor) showed a different ability to induce pancreatic insulin secretion *in vitro* [40]. Consistent with our findings in obese older adults, DI followed a similar trend as that of insulin sensitivity [13] and the inclusion of circulating adipocytokines in our model, accounted for higher increase in the predictability of DI variance in the diet-exercise group compared to that in the diet group. Furthermore, a significant positive correlation was observed between DI and measures of physical function (VO_2peak_ and muscle strength) in both the diet and diet-exercise groups. Physical function provided a higher predictability of DI in the diet-exercise group, consistent with data describing a positive association between exercise-induced improvement in fitness and *β*-cell function [36]. VO_2peak_ reflects mitochondrial oxidation capacity, and its improvement in our population (best in the diet-exercise group [9]) could reduce *β*-cell lipotoxicity and glucotoxicity [30]. Moreover, exercise-induced increase in VO_2peak_ was shown to improve insulin signaling and mitochondrial respiration in rats' pancreatic cells [13, 41]. The existing evidence of an inverse relation between physical fitness (defined by VO_2peak_ and max leg power press) and oxidative stress [42] led us to speculate that improvements in VO_2peak_ in our population of frail, obese older adults could reduce the oxidative stress, typical of this condition, and perhaps reduce pancreas toxicity. It is possible that diet-induced weight loss is a prerequisite necessary to experience some metabolic benefits induced by exercise, explaining the lack of DI changes in our exercise group [13].

The strengths of our study include the degree of adherence to the RCT which facilitated the assessment of the distinct effects of each lifestyle intervention. The metabolic assessments performed 48 to 72 h after the last bout of exercise allowed us to examine the chronic rather than the acute effects of the interventions. The similar degree of weight loss in the diet and diet-exercise groups allowed for unbiased comparisons, and the repeated measures at several time points allowed for the examination of the temporal pattern of changes over time. Limitations include the relatively small sample size and the lack of adjustment for vitamin K known to impact OC carboxylation. Another limitation is that we did not have data on the influence of different types of food in relation to UcOC.

## 5. Conclusions

This is the first RCT investigating the long-term effect of different lifestyle interventions on UcOC and DI in frail, obese older adults. Our findings suggest that UcOC can contribute to the metabolic adaptation to caloric restriction only in conditions of active weight loss and when not accompanied by exercise. On the other hand, diet and regular exercise may have additive effects on insulin secretion when lifestyle intervention is sustained. Our results provide evidence that diet and diet-exercise may improve insulin secretion through different mechanisms: diet through changes in UcOC and body composition while diet-exercise through changes in circulating adipocytokines and physical function. These metabolic effects are likely to reduce the risk of developing T2D and other metabolic abnormalities in this population.

## Figures and Tables

**Table 1:** Baseline characteristics of participants.

	Control (*n* = 27)	Diet (*n* = 26)	Exercise (*n* = 26)	Diet-exercise (*n* = 28)	*P* value^†^
Age (y)	69 ± 1	70 ± 1	70 ± 1	70 ± 1	0.85
Female/male (*n*)	18/9	17/9	16/10	16/12	0.89
White, *n* (%)	22 (81)	23 (88)	21 (81)	25 (89)	0.78
Height (cm)	165.8 ± 1.9	169.2 ± 1.9	168.1 ± 1.1	165.4 ± 1.6	0.38
Weight (kg)	101.0 ± 3.2	104.1 ± 2.9	99.2 ± 3.3	99.1 ± 3.4	0.66
Body mass index (kg/m^2^)	37.3 ± 0.9	37.2 ± 0.9	36.9 ± 1.1	37.2 ± 1.0	0.93
Body composition					
Fat-free mass (kg)	57.3 ± 2.2	61.4 ± 2.5	57.6 ± 2.7	57.2 ± 1.9	0.17
Fat mass (kg)	43.8 ± 1.9	42.8 ± 1.3	41.6 ± 1.9	41.9 ± 2.2	0.84
Trunk fat (kg)	22.4 ± 1.1	21.8 ± 0.8	21.2 ± 0.8	20.9 ± 0.9	0.54
Bone turnover and BMD					
CTX (ng/ml)	0.407 ± 0.039	0.309 ± 0.024	0.350 ± 0.029	0.320 ± 0.023	0.09
PINP (*μ*g/l)	52.2 ± 5.3	41.5 ± 2.4	45.2 ± 2.8	43.1 ± 2.5	0.14
WB BMD (g/cm^2^)	1.207 ± 0.034	1.252 ± 0.036	1.188 ± 0.034	1.269 ± 0.033	0.30
Physical function					
Knee extension strength (Nm)	70.0 ± 5.2	70.8 ± 4.8	72.6 ± 5.6	71.4 ± 4.5	0.99
Knee flexion strength (Nm)	44.7 ± 3.6	50.3 ± 3.5	46.4 ± 3.4	49.1 ± 2.8	0.63
VO_2peak_ (l/min)	1.7 ± 0.1	1.8 ± 0.1	1.8 ± 0. 1	1.7 ± 0.1	0.66
Adipocytokines					
hs-CRP (mg/l)	22.4 ± 1.1	21.8 ± 0.8	21.2 ± 0.8	20.9 ± 0.9	0.72
sTNF R1 (pg/ml)	167.2 ± 7.6	155.5 ± 6.9	166.6 ± 10.0	177.5 ± 9.4	0.36
Interleukin 6 (pg/ml)	3.1 ± 0.8	1.8 ± 0.1	1.7 ± 0.2	2.6 ± 0.7	0.15
Leptin (*μ*g/l)	38.1 ± 5.2	36.7 ± 3.4	37.5 ± 5.2	34.1 ± 3.9	0.92
Adiponectin (ng/ml)	31.9 ± 4.7	23.3 ± 2.4	20.8 ± 1.5	24.4 ± 2.5	0.06

WB BMD: whole body bone mineral density; CTX: C-terminal telopeptide of type I collagen; PINP: intact N-terminal propeptide of type I procollagen; VO_2peak_: peak oxygen uptake; hs-CRP: high-sensitivity C-reactive protein; sTNF R1: soluble tumor necrosis factor receptor 1. Values are given as mean ± SE. ^†^*P* values as calculated with the use of analyses of variance for quantitative data and chi-square tests for counts.

**Table 2:** Effect of diet, exercise, or both on undercarboxylated osteocalcin, insulin secretion, and insulin sensitivity^∗^.

Outcome variables	Control (*n* = 27)	Diet (*n* = 26)	Exercise (*n* = 26)	Diet-exercise (*n* = 28)	*P* value^†^				
					Diet versus control	Exercise versus control	Diet-exercise versus control	Diet-exercise versus diet	Diet-exercise versus exercise
UcOC (ng/ml)									
Baseline	4.7 ± 0.6	3.9 ± 0.4	4.3 ± 0.6	3.6 ± 0.4					
Change at 6 months	−0.2 ± 0.5	1.1 ± 0.4^¶^	0.2 ± 0.5	−0.1 ± 0.4					
Change at 1 year	−0.7 ± 0.5	1.4 ± 0.5^¶^	0.2 ± 0.5	0.4 ± 0.4	0.007	0.27	0.11	0.20	0.67
Total OC (ng/ml)									
Baseline	12.4 ± 1.0	11.4 ± 0.8	13.2 ± 0.9	12.2 ± 0.8					
Change at 6 months	−0.8 ± 0.6	2.5 ± 0.6^‡^	−1.4 ± 0.6^¶^	−0.3 ± 0.5					
Change at 1 year	−0.8 ± 0.6	2.0 ± 0.6^§^	−1.8 ± 0.6^¶^	0.5 ± 0.5	0.006	0.29	0.18	0.12	0.01
UcOC/total OC									
Baseline	0.42 ± 0.06	0.40 ± 0.05	0.33 ± 0.04	0.33 ± 0.04					
Change at 6 months	−0.01 ± 0.07	0.07 ± 0.06	0.01 ± 0.04	−0.01 ± 0.06					
Change at 1 year	−0.02 ± 0.05	0.10 ± 0.04	0.11 ± 0.05	0.04 ± 0.06	—	—	—	—	—
IGI									
Baseline	1.2 ± 0.2	1.4 ± 0.2	1.5 ± 0.2	1.2 ± 0.2					
Change at 6 months	0.1 ± 0.1	0.2 ± 0.2	0.1 ± 0.2	0.3 ± 0.1					
Change at 1 year	−0.2 ± 0.4	0.0 ± 0.2	−0.1 ± 0.2	0.3 ± 0.1	—	—	—	—	—
ISI									
Baseline	3.3 ± 0.4	2.7 ± 0.3	3.5 ± 0.5	3.4 ± 0.5					
Change at 6 months	−0.1 ± 0.3	1.1 ± 0.3^‡^	0.1 ± 0.3	1.2 ± 0.2^§^					
Change at 1 year	0.2 ± 0.3	1.2 ± 0.3^‡^	0.1 ± 0.3	2.4 ± 0.3^‡^	0.04	0.73	<0.001	0.02	<0.001
DI									
Baseline	3.0 ± 0.3	3.1 ± 0.4	4.0 ± 0.7	3.8 ± 0.6					
Change at 6 months	0.1 ± 0.4	2.1 ± 0.5^§^	0.1 ± 0.5	1.8 ± 0.4^¶^					
Change at 1 year	−0.1 ± 0.5	1.9 ± 0.5^§^	0.2 ± 0.5	3.5 ± 0.4^‡^	0.02	0.67	<0.001	0.04	0.001

UcOC: undercarboxylated osteocalcin; OC: osteocalcin; IGI: insulinogenic index; ISI: insulin sensitivity index; DI: disposition index. ^∗^Change scores are the least square adjusted means ± SE from the repeated measures analyses; baseline values are the observed means ± SE. ^†^*P* values for the comparison among the groups of changes from baseline to 6 months were calculated with the use of mixed model repeated measures analyses of variance (with baseline values and sex as covariates) and are reported when the overall *P* value was less than .05 for the interaction among the 4 groups over time. *P* values for the group × time interaction were .04 for UcOC, <0.001 for total OC, <0.001 for ISI, and <0.001 for DI. ^‡^*P* < 0.001, ^§^*P* < 0.01, and ^¶^*P* < .05 for the comparison of the value at the follow-up time with the baseline value within the group, as calculated with the use of mixed model repeated measures analysis of variance.

**Table 3:** Hierarchical models of changes in undercarboxylated osteocalcin.

	*R*	*R* ^2^	Δ*R*^2^	*B*	SE	*β*	*P*
*Diet group*							
Model 1	.526	.276^†^					
Change in PINP				.090	0.047	0.321	0.066
Change in CTX				.006	0.004	0.266	0.141
Change in WB BMD				−16.5	13.390	−0.197	0.226
Model 2	.659	.434^†^	.158^∗^				
Change in PINP				−0.118	0.049	0.548	0.004
Change in CTX				−0.003	0.006	−0.121	0.610
Change in WB BMD				−27.669	12.865	−0.330	0.038
Change in FFM				7.78*E*−5	0.0001	0.083	0.742
Change in FM				−0.0002	0.0002	−0.651	0.013
Model 3	.848	.719^∗^	.284^†^				
Change in PINP				0.135	0.048	0.481	0.009
Change in CTX				0.015	0.006	0.652	0.023
Change in WB BMD				−15.880	11.490	−0.190	0.177
Change in FFM				−6.350*E*−5	0.0001	−0.068	0.772
Change in FM				−0.0001	0.0002	−0.239	0.553
Change in hs-Crp				−1.293	0.349	−0.707	<0.001
Change in IL-6				−4.333	0.903	−0.719	<0.001
Change in sTNF R1				0.024	0.021	0.205	0.251
Change in leptin				0.084	0.059	0.478	0.165
Change in adiponectin				−0.351	0.091	−0.644	<0.001
*Diet-exercise group*							
Model 1	.706	.498^∗^					
Change in PINP				0.053	0.024	0.319	0.032
Change in CTX				0.008	0.002	0.460	0.003
Change in WB BMD				−9.324	9.538	−0.107	0.334
Model 2	.708	.501^∗^	.003				
Change in PINP				0.048	0.026	0.287	0.077
Change in CTX				0.008	0.003	0.486	0.005
Change in WB BMD				−8.395	10.573	−0.096	0.432
Change in FFM				−8.502*E*−5	0.0001	−0.72	−0.665
Change in FM				4.265*E*−5	0.0001	−0.88	0.611
Model 3	.783	.613^∗^	.112				
Change in PINP				0.103	0.031	0.625	0.002
Change in CTX				0.003	0.003	0.192	0.309
Change in WB BMD				−34.315	13.510	−0.392	0.016
Change in FFM				−0.0002	0.0002	−0.251	0.156
Change in FM				−0.0002	0.0001	−0.480	0.088
Change in hs-Crp				0.194	0.109	0.283	0.084
Change in IL-6				−0.091	0.393	−0.035	0.818
Change in sTNF R1				0.017	0.014	0.184	0.234
Change in leptin				0.133	0.055	0.639	0.022
Change in adiponectin				0.077	0.042	0.275	0.077

PINP: intact N-terminal propeptide of type I procollagen; CTX: C-terminal telopeptide of type I collagen; WB BMD: whole body bone mineral density; FFM: fat-free mass; FM: fat mass; hs-CRP: high-sensitivity C-reactive protein; IL6: interleukin 6; sTNF R1: soluble tumor necrosis factor receptor 1. ^∗^Values are given as mean ± SE. ^∗^*P* < .001; ^†^*P* < .01.

**Table 4:** Hierarchical models of changes in disposition index.

	*R*	*R* ^2^	Δ*R*^2^	*B*	SE	*β*	*P*
*Diet group*							
Model 1	.449	.202^†^					
Change in UcOC				0.349	0.119	0.448	0.006
Model 2	.844	.712^∗^	.511^†^				
Change in UcOC				0.281	0.076	0.361	<0.001
Change in FFM				0.001	0.0001	1.042	<0.001
Change in FM				−0.0004	0.0001	−1.301	<0.001
Model 3	.923	.810^∗^	.140^∗^				
Change in UcOC				0.188	0.086	0.241	0.037
Change in FFM				0.001	0.0001	1.149	<0.001
Change in FM				−0.0004	0.0001	−1.262	<0.001
Change in hs-Crp				−0.517	0.352	−0.300	0.154
Change in IL-6				−1.648	0.507	−0.356	0.003
Change in sTNF R1				0.010	0.010	0.108	0.348
Change in leptin				0.010	0.030	0.074	0.744
Change in adiponectin				0.085	0.057	0.176	0.146
Model 4	.967	.935^∗^	.083^†^				
Change in UcOC				−0.037	0.088	−0.048	0.675
Change in FFM				0.001	0.0001	0.891	<0.001
Change in FM				−0.0003	0.0001	−1.100	<0.001
Change in Hs-Crp				0.096	0.359	0.056	0.791
Change in IL-6				−2.234	0.427	−0.483	<0.001
Change in sTNF R1				−0.12	0.010	−0.127	0.250
Change in leptin				0.022	0.028	0.165	0.432
Change in adiponectin				0.110	0.045	0.228	0.022
Change in KE strength				−0.161	0.033	−0.558	<0.001
Change in KF strength				0.109	0.024	0.344	<0.001
Change in VO_2peak_				0.372	0.130	0.407	0.009
*Diet-exercise group*							
Model 1	.278	.078					
Change in UcOC				0.679	0.403	0.278	0.101
Model 2	.525	.276^†^	.198^†^				
Change in UcOC				0.544	0.369	0.222	0.150
Change in FFM				0.0004	0.001	0.180	0.387
Change in FM				−0.001	0.0002	−0.554	0.011
Model 3	.838	.702^∗^	.427^†^				
Change in UcOC				0.016	0.285	0.006	0.956
Change in FFM				0.001	0.0002	0.510	0.006
Change in FM				−0.0003	0.0004	−0.359	0.106
Change in hs-Crp				0.108	0.208	0.074	0.608
Change in IL-6				−0.537	0.871	−0.086	0.543
Change in TNF-R				−0.038	0.034	−0.180	0.267
Change in leptin				−0.074	0.115	−0.148	0.526
Change in adiponectin				0.475	0.101	0.686	<0.001
Model 4	.972	.946^∗^	.243^†^				
Change in UcOC				−1.269	0.221	−0.518	<0.001
Change in FFM				0.002	0.0002	0.658	<0.001
Change in FM				0.001	0.0002	1.318	<0.001
Change in Crp				0.149	0.096	0.102	0.132
Change in IL-6				0.096	0.422	0.015	0.822
Change in sTNF R1				−0.077	0.019	−0.362	<0.001
Change in leptin				−0.396	0.062	−0.793	<0.001
Change in adiponectin				0.495	0.058	0.715	<0.001
Change in KE strength				0.191	0.038	0.423	<0.001
Change in KF strength				0.339	0.052	0.434	<0.001
Change in VO_2peak_				1.628	0.319	0.909	<0.001

UcOC: undercarboxylated osteocalcin; FFM: fat-free mass; FM: fat mass; hs-CRP: high-sensitivity C-reactive protein; IL6: interleukin 6; sTNF R1: soluble tumor necrosis factor receptor 1; KE: knee extension; KF: knee flexion. ^∗^Values are given as mean ± SE. ^∗^*P* < .001; ^†^*P* < .01.
